# Ketonization of Ginsenoside C-K by Novel Recombinant 3-β-Hydroxysteroid Dehydrogenases and Effect on Human Fibroblast Cells

**DOI:** 10.3390/molecules28093792

**Published:** 2023-04-28

**Authors:** Yan Jin, Dandan Wang, Wan-Taek Im, Muhammad Zubair Siddiqi, Deok-Chun Yang

**Affiliations:** 1 School of Life Science, Nantong University, Nantong 226019, China; jinyan224@hotmail.com; 2 College of Life Sciences, Yantai University, Yantai 264005, China; whdandan@163.com; 3 Department of Biotechnology, Hankyong National University, 327 Jungang-ro, Anseong-si 17579, Gyeonggi-do, Republic of Korea; wandra@hknu.ac.kr; 4 Department of Oriental Medicinal Material & Processing, College of Life Science, Kyung Hee University, Seocheon-dong, Giheung-gu, Yongin-si 17104, Gyeonggi-do, Republic of Korea

**Keywords:** *Lactobacillus brevis* DCY65-1, 3-β-hydroxysteroid dehydrogenase, ketonization, ginsenoside C-K, 3-oxo-C-K

## Abstract

Background and objective: The ginsenoside compound K (C-K) (which is a de-glycosylated derivative of major ginsenosides) is effective in the treatment of cancer, diabetes, inflammation, allergy, angiogenesis, aging, and has neuroprotective, and hepatoprotective than other minor ginsenosides. Thus, a lot of studies have been focused on the conversion of major ginsenosides to minor ginsenosides using glycoside hydrolases but there is no study yet published for the bioconversion of minor ginsenosides into another high pharmacological active compound. Therefore, the objective of this study to identify a new gene (besides the glycoside hydrolases) for the conversion of minor ginsenosides C-K into another highly pharmacological active compound. Methods and Results: *Lactobacillus brevis* which was isolated from Kimchi has showed the ginsenoside C-K altering capabilities. From this strain, a novel potent decarboxylation gene, named HSDLb1, was isolated and expressed in *Escherichia coli* BL21 (DE3) using the pMAL-c5X vector system. Recombinant HSDLb1 was also characterized. The HSDLb1 consists of 774 bp (258 amino acids residues) with a predicted molecular mass of 28.64 kDa. The optimum enzyme activity was recorded at pH 6.0–8.0 and temperature 30 °C. Recombinant HSDLb1 effectively transformed the ginsenoside C-K to 12-β-hydroxydammar-3-one-20(S)-O-β-D-glucopyranoside (3-oxo-C-K). The experimental data proved that recombinant HSDLb1 strongly ketonized the hydroxyl (-O-H) group at C-3 of C-K via the following pathway: C-K → 3-oxo-C-K. In vitro study, 3-oxo-C-K showed higher solubility than C-K, and no cytotoxicity to fibroblast cells. In addition, 3-oxo-C-K induced the inhibitory activity of ultraviolet A (UVA) against matrix metalloproteinase-1 (MMP-1) and promoted procollagen type I synthesis. Based on these expectations, we hypothesized that 3-oxo-C-K can be used in cosmetic products to block UV radiations and anti-ageing agent. Furthermore, we expect that 3-oxo-C-K will show higher efficacy than C-K for the treatment of cancer, ageing and other related diseases, for which more studies are needed.

## 1. Introduction

In eastern Asia, *Panax ginseng* (family *Araliaceae*) is a well-known natural and traditional medicine, also known as the “king of herbs” [1]. The root of *Panax ginseng* consists of 80–90% organic and inorganic substances, along with several active saponins, carbohydrates, vitamins, peptides, nitrogenous substances, minerals and essential oils [2]. Among them, saponins are commonly known as major ginsenosides (Rb1, Rc, Rb2, Rb3, Rd, Re and Rg1). The major ginsenosides are the bioactive constituents of *Panax ginseng* which are triterpene and used as marker compounds for the identification of ginseng species [3]. Based on hydroxyl group position, the major ginsenosides are classified into three broad classes: protopanaxadiols (PPD), protopanaxatriols (PPT), and oleanic acid type ginsenosides [4]. More than 90% of ginseng extract contain PPD (Rb1, Rb2, Rc and Rd) and PPT (Rg1 and Re) type major ginsenosides, whereas the minor ginsenosides account for ≤0.2%, including C-K, Rg3, Rg5, Rh2, Rk1, Rh1, and Rg2 [5,6,7]. As shown in previous studies that major ginsenosides are poorly absorbed by human gastrointestinal track due to their high molecular weight [4,5,6,7]. Therefore, these major ginsenosides are transformed into minor ginsenosides, which show a good permeability across cell membranes and bioavailability. Prior studies have demonstrated that minor ginsenosides (Rg3, Rh2, F2, Rk1, Rg5, Rh1, and C-K) are pharmacological more active than major ginsenosides [8,9,10,11,12,13,14] and derived from the major ginsenosides using three different conversion processes including biotransformation (microbial or enzyme), chemical (acid or base) and physical (heat) transformation [15,16]. But nowadays, the enzymatic (recombinant enzyme) transformation of major ginsenosides to minor ginsenosides is known as one of the most suitable technology for obtaining of target minor ginsenosides due to its control environmental condition as compared to physical and chemical conversion treatment. Microbial or enzymatic modification of ginsenosides includes ketonization, hydroxylation, and side-chain oxidation–reduction [15,16,17,18,19]. Therefore, the awareness of researchers in medicinal use of minor ginsenosides is increasing due to its high pharmacological effects [8,9,10,11,12,13,14,16]. 

Among the minor ginsenosides (Rg3, Rh2, F2, Rk1, Rg5, Rh1, and C-K), the C-K is well studied for the treatment of cancer, thrombosis, diabetes, photoaging, inflammation, oxidation, and improvement of the immune system [20,21,22,23,24]. In addition, some relevant studies evaluated the effect of minor ginsenosides (including C-K C-Y and C-Mx,) on skin aging and collagen degradation by regulation of MMP1 and COX-2 expression under ultraviolet (UV) irradiation conditions [25,26,27]. Due to the high pharmacological effects of C-K, it can be obtained via bioconversion of low active major ginsenosides Rb1, Rb2, Rb3, Rc, and Rd using both glycoside hydrolase positive bacterial strains, or recombinant glycosidases [15,16,17,27]. Extensive research has been carried out for the conversion of major ginsenosides to minor ginsenosides to enhance the efficacy, but there is no single study exists for the further conversion of C-K (by using recombinant enzyme) to another high pharmacological active compound. Though, it was reported previously that *Lactobacillus brevis* DCY65-1 was positive for the ketonization (a reaction in which a compound alters into a ketone compound) of minor ginsenoside F1 → 6α,12β-dihydroxydammar-3-one-20(*S*)-*O*-β-d-glucopyranoside and C-K → 12β-hydroxydammar-3-one-20(*S*)-*O*-β-d-glucopyranoside [17]. But, the study failed to find out the target ketonizing gene. Therefore, our research team finds out a novel 3-β-hydroxysteroid dehydrogenase gene from *L. brevis* DCY65-1. The novel 3-β-hydroxysteroid was responsible for the bioconversion or ketonization of C-K. 

Hydroxysteroid dehydrogenases (HSDs) are key enzymes that catalyze the dehydrogenation of hydroxysteroids. HSD enzymes belong to the short-chain dehydrogenases/reductases (SDR) or aldo-keto reductases (AKR) superfamily [28]. HSD enzymes are classified into four groups; 3-β-hydroxysteroid dehydrogenases, 11-β-hydroxysteroid dehydrogenases, 17-β-hydroxysteroid dehydrogenases, and 20-α-β-hydroxysteroid dehydrogenases [28]. Each group of these HSDs [3-β-, 11-β-, 17-β-, and 20-α-β-hydroxysteroid dehydrogenases] has different tissue localization and functions. The 3-β-HSDs (EC 1.1.1.51) are the significant class of enzyme that can modify the hydroxyl groups (-O-H) on steroids or 3-oxo derivatives. These small modifications in the structure of a compound greatly affect its solubility, cytotoxicity, and ability to activate or inhibit host receptors [29,30,31,32]. Because of high functional activity of 3-β-HSDs, several 3-β-HSDs have been isolated from different sources including higher plants [33], animals and bacteria [34,35,36,37]. However, there is no single study exists for the structure modification of minor ginsenosides to 3-oxo derivatives using recombinant hydroxysteroid dehydrogenases. 

This is the first study in which a novel ketonizing gene (*HSDLb1*) was isolated from *L. brevis* DCY65-1 and cloned in *E*. *coli* BL21. The expressed ketonizing enzyme (HSDLb1) was positive for the ketonization of the hydroxyl group at the C-3 position of C-K and effectively converted C-K to 3-oxo-C-K. In addition, the pharmacological activity of 3-oxo-C-K was evaluated against the ultraviolet (UV) damaged human dermal fibroblast cells by measuring the metalloproteinase-1 (MMP-1) expression and procollagen type I production. Therefore, we aimed that the 3-oxo-C-K may be a pharmacologically more active compound than C-K for which further study (both in vitro and in vivo) will be needed.

## 2. Results and Discussion

### 2.1. Bacterial Strain Isolation and Screening for Ketonzation of C-K

The strain DCY65-1 was isolated from Kimchi after 7 days of incubation on MRS agar medium at 37 °C. The strain DCY65-1 was positive for the initial bioconversion of C-K to 3-oxo-C-K [17]. In addition, the target gene [HSDLb1 (3-β-HSD gene)] was identified and cloned for further analysis. 

### 2.2. Phylogenetic Analysis

HSDLb1 (3-β-HSD gene) consists of 774 nucleotides or 258 amino acids with a molecular mass of 28.64 kDa. The theoretical pI value of HSDLb1 is 5.30 (http://web.expasy.org/compute_pi/) (accessed on 27 January 2023). HSDLb1 has homology to the protein domain of 3-β-HSD and belonging to short-chain dehydrogenases/reductases (SDR) family using interPro online sequence analysis (https://www.ebi.ac.uk/interpro) (accessed on 27 January 2023). The coding sequence of protein HSDLb1 (HSDLb1 cds) was deposited at DDBJ/EMBL/GenBank under accession number OP681430. A phylogenetic tree was constructed using 3-β-HSD sequences from various sources including, human, rat, austern (*Crassostrea angulate*), mice, *L. brevis*, chicken, frog, and catfish as shown in Figure S1. Additionally, Figure S1 also shows that 3-β-HSD genes were also isolated from different sources including plants, mice, and bacteria, which show NAD(P)-binding Rossmann-fold domains as homologous super family (https://www.ebi.ac.uk/interpro) (accessed on 27 January 2023).

### 2.3. Expression and Purification of Recombinant HSDLb1

**Figure 1:**
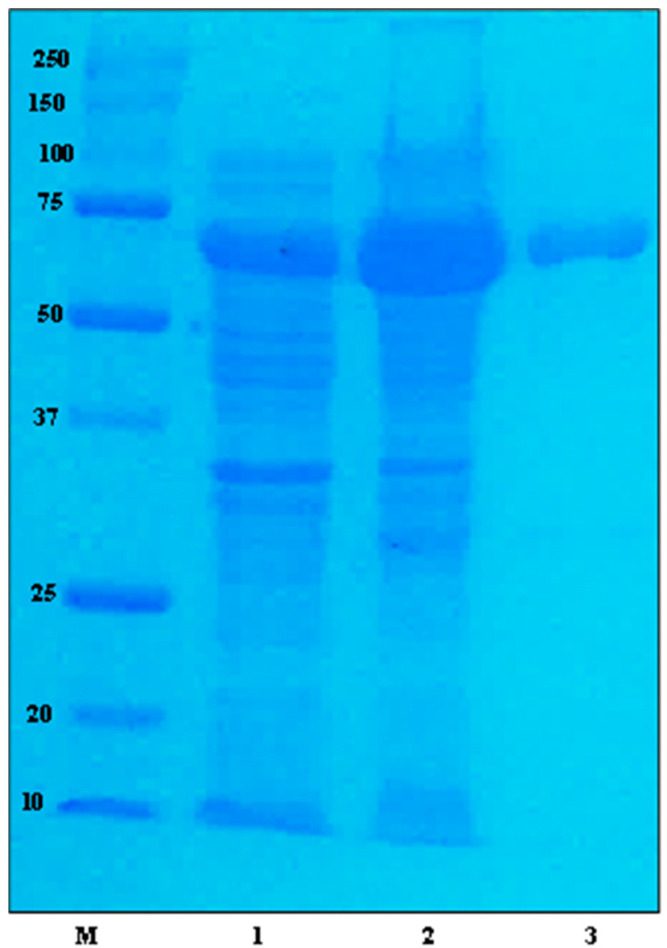
SDS-PAGE analysis of the recombinant HSDLb1. M, molecular mass marker (10–250 kDa, Ppromega); 1, uninduced crude extract recombinant enzyme (control); 2, induced crude extract of recombinant BL21 (DE3) cells carrying pMAL-HSDLb1; 3, pMAL-HSDLb1 after purification with the MBP-bind agarose resin.

The amplification of HSDLb1 was done by polymerase chain reaction (PCR) and then introduced into the pMAL-c5X vector system and expressed in *E. coli*. The expressed protein was purified [MBP•bind agarose resin was used to purify the recombinant enzyme, (MBP-HSDLb1) as described previously, [16] and analyzed using SDS-PAGE (Figure 1). The molecular mass of MBP-HSDLb1 (the molecular mass of MBP + molecular mass of HSDLb1) calculated by the amino acid sequence was 71.15 kDa, which is similar to the molecular mass identified by SDS-PAGE analysis (Figure 1).

### 2.4. Characterization of Recombinant HSDLb1

**Figure 2:**
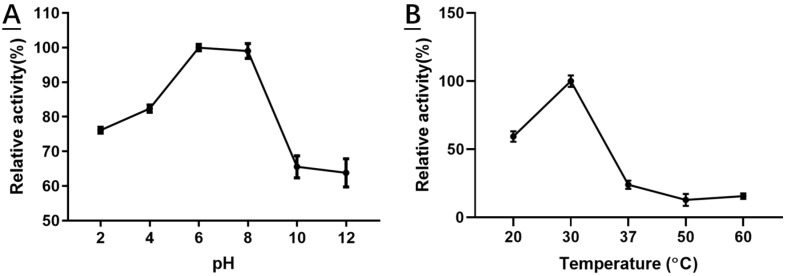
Effects of pH (**A**) and temperature (**B**) on the stability and activity of recombinant HSDLb1. Both pH and temperature experiments were carried out in triplicate.

HSDLB1 was active between pH values of 2.0–9.0 at 30 °C, but the optimum activity was recorded at pH 6.0–8.0 in sodium phosphate buffer (Figure 2A). The enzyme lost more than 35% of its optimal activity when the pH was >8.0. In addition, a rise in temperature values strongly affected the HSDLB1 activity. The optimal temperature activity for HSDLb1 was 30 °C (Figure 2B). However, HSDLb1 lost more than 65% of its activity at 37 °C and 90% of its activity at 50 °C (Figure 2B). 

Typically, the enzymes activities are very sensitive to high concentrations of metals ions, therefore, the effects of metal ions (final concentration of 10 mM) on HSDLb1 activity were investigated. HSDLb1 activity was not affected significantly by NaCl, KCl, NH_4_Cl and CaCl_2_ (Table 1), but Mg^2+^ boosted the HSDLb1 activity. Additionally, the HSDLb1 activity was significantly inhibited in the presence of 10 mM Co^2+^, Cu^2+^, Fe^3+^, and Zn^2+^ (Table 1).

### 2.5. Bioconversion of Ginsenoside C-K by Recombinant HSDLb1

**Figure 3:**
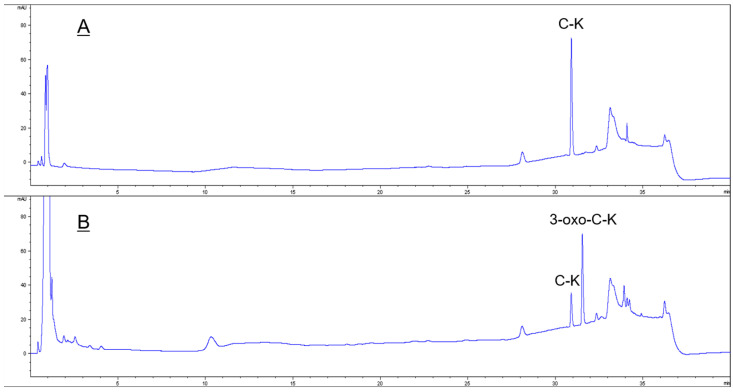
HPLC profiles of metabolites of ginsenoside C-K converted by recombinant HSDLb1. (**A**) ginsenoside C-K (as a control); (**B**) transformed C-K metabolite.

**Figure 4:**
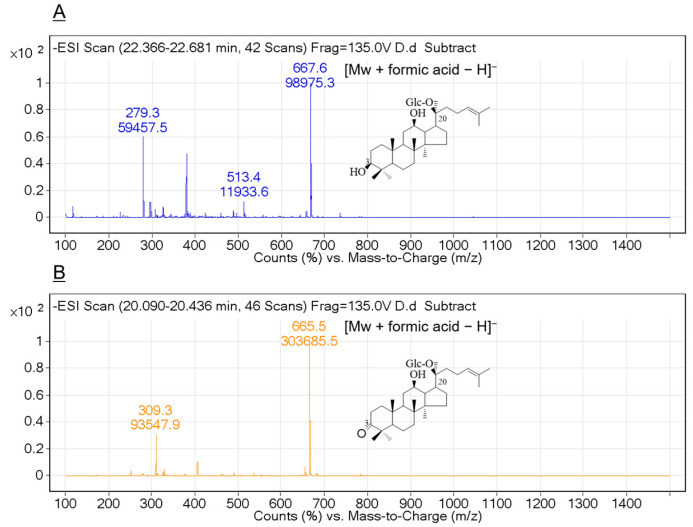
Mass spectra of ginsenoside C-K converted by recombinant HSDLb1. (**A**) Mass spectrum of C-K, *m*/*z* 667.6 = [Mw + formic acid − H]^−^, Mw 622.9; (**B**) Mass spectrum of 3-oxo-C-K, *m*/*z* 665.5 = [Mw + formic acid − H]^−^, Mw 620.5.

To confirm the biotransformation pathway of ginsenoside C-K using recombinant HSDLb1, TLC, HPLC and LC/MS analyses were performed after 24 h incubation of the sample at its optimum temperature and pH. Based on the Rf value, it is clear that recombinant HSDLb1 transforms C-K to 3-oxo-C-K after 48 h of incubation at 30 °C. The HPLC results for the reaction mixture of C-K and transformed C-K (3-oxo-C-K) are shown in Figure 3A,B. The HPLC peaks of C-K and 3-oxo-C-K were identified from their retention times after comparison with ginsenoside standards and control as previously described [28]. Figure 3A was the HPLC result of C-K before biotransformation, and Figure 3B was the HPLC result of the product transformed by HSDLB1. These results showed that C-K is converted to 3-oxo-C-K by HSDLB1. Additionally, to determine the molecular weights (Mw) of C-K and 3-oxo-C-K, the samples were subjected to LC/MS analysis. The LC/MS showed a pseudomolecular peak [M + formic acid − H]^−^ at *m*/*z* 667.6 in MS-ESI, equivalent to the molecular weight of ginsenoside C-K [C_36_H_62_O_8_ (calculated molecular weight, 622.9)] as shown in Figure 4A. Similarly, the produced molecular weight of 3-oxo-C-K was identified as C_36_H_60_O_8_ based on the protonated molecular ion peak [M + formic acid − H]^−^ at *m*/*z* 665.5 (Mw, 620.5) in MS-ESI analysis. The LC/MS results confirmed the conversion of ginsenoside C-K to 3-oxo-C-K (Figure 4B).

**Figure 5:**
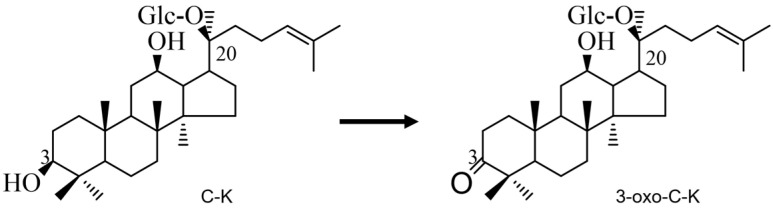
Microbial ketonization pathway of ginsenoside C-K by recombinant HSDLb1. 1, C-K; 2, 3-oxo-C-K.

As a result of the analytical analysis (TLC, HPLC and LC/MS), the bioconversion pathway for the C-K was suggested as C-K → 3-oxo-C-K (Figure 5). Thus, on the basis of the data presented here, it was confirmed that the recombinant HSDLb1 has potent ketonic decarboxylase activity of C-K into 3-oxo-C-K by cutting the –O-H group attached at the C-3 position of C-K. Thus, 3β-HSD or recombinant HSDLb1 derived from *L. brevis* DCY65-1 may be a new enzyme that converts the minor ginsenoside C-K via a pathway comparable to progesterone biosynthesis in the adrenal gland [32]. This is the first report of the biosynthesis of a ketonized compound (3-oxo-C-K) by using the 3β-HSD gene from *L. brevis* DCY65-1. In the GenBank the enzyme coding sequence was submitted as the synonym *Levilactobacillus brevis*.

### 2.6. Cell Cytotoxicity

#### 2.6.1. Effect of C-K and 3-oxo-C-K on HDF Cell Viability

**Figure 6:**
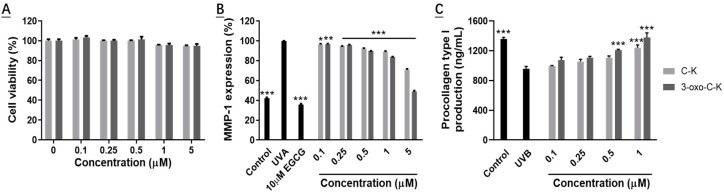
Effect of C-K and 3-oxo-C-K on MPP-1 and procollagen type 1 secretion in UV-irradiated HDFs. Fibroblast cells were pre-incubated with or without compounds for 24 h, and cell viability was evaluated using MTT assay. Data represent the mean ± SD (standard deviation) of triplicate experiments (**A**). Cells were non-irradiated or irradiated with UV, followed by treatment with the different concentrations of 3-oxo-C-K for 24 h. Production of MMP-1 (**B**), and procollagen type 1 (**C**) under non-UV irradiation and UV-irradiated conditions. Epigallocatechin gallate (EGCG) was used as a positive control. The results were expressed as the average ± SD of triplicate determinations. * *p* < 0.01, ** *p* < 0.01 and *** *p* < 0.001 compared with UVA/UVB irradiation. Each experiment was carried out in triplicate.

To identify the cytotoxicity of C-K and 3-oxo-C-K, HDFs were treated with different concentrations of C-K and 3-oxo-C-K. Both C-K and 3-oxo-C-K did not disturb the cell viability of HDFs until the concentration reached 5 µM and the cell viability was almost similar to that of control (Figure 6A). Cell viability decreased with increasing concentration of C-K and 3-oxo-C-K, but there was no significant difference. As described previously that more than 80% of the cell viability is considered to be non-cytotoxic [24]. Previous studies have shown that C-K is not cytotoxic [38,39]. Therefore, these results showed that 3-oxo-C-K was not significantly toxic for the skin cells.

#### 2.6.2. Effect of C-K and 3-oxo-C-K on the Synthesis of MMP-1 and Procollagen Type I 

Skin aging occurs because of collagen degradation via the indication of MMPs by UV irradiation, decreased collagen synthesis and increased levels of MMP-1, and these changes lead to changes such as wrinkling and decreased elasticity of the skin. To investigate the effects of C-K and 3-oxo-C-K on UV-induced damage cells, the levels of MMP-1, and procollagen type I in the supernatants, the samples were measured by ELISA kits [40,41,42]. Compared with the control group, UV irradiation increased MMP-1 expression 2.36 times. The inhibition rate of MMP-1 increased with the increase of C-K and 3-oxo-C-K concentrations. At the concentration of 5 μM, the inhibition rates of C-K and 3-oxo-C-K were 28.72 and 50.82%, respectively. Results showed that 3-oxo-C-K (5 μM) suppressed UV-induced MMP-1 more than C-K (Figure 6B). Epigallocatechin gallate (EGCG) was used as a positive control. On the other hand, procollagen type I was reduced by 29.41% through UV irradiation compared to the control group. 1 μM 3-oxo-C-K increased procollagen type I by 1.43 times, which was not significantly different with the control group. Procollagen type I is an important component of the skin dermis. Therefore, our results show that 3-oxo-C-K (1 μM) treatment increases the secretion of proteins related to collagen synthesis (procollagen type I) (Figure 6C).

As C-K is considered a good pharmacological agent for the treatment of cancer, thrombosis, diabetes, ageing, inflammation, oxidation, skin dehydration or photoaging, and improving immune system [8,9,10,11,12,13,14], and therefore, it can be obtained via bioconversion (use of bacterial enzymes or recombinant enzymes) of major ginsenosides Rb1, Rc and Rd. Thus, the alteration of ginsenosides increases the efficacy, and therefore, further conversion of C-K will lead a new drug discovery in a systemically related disease. 

Enzymatic fermentation methods for the transformation of ginsenosides produce good ginseng products with almost no by-products. Therefore, in this study a novel ketonizing gene (HSDLb1) was isolated from L. brevis DCY65-1, which is responsible for the ketonization of ginsenoside C-K, was cloned and expressed in E. coli. The HSDLb1 consists of 774 nucleotides or 258 amino acids residues with molecular mass of 28.64 kDa (identified via amino acid sequence analysis). The recombinant HSDLb1 showed optimal enzyme activity at pH 6–8 and temperature 30 °C. In addition to the C-K transformation ability of HSDLb1, we found that 10 mM MgCl_2_ boosted the enzyme activity. In addition, the NaCl, NH_4_Cl, KCl, and CaCl_2_, weakly effected the activity of the recombinant HSDLb1, but 10 mM CoCl_2_, FeCl_3_, CuSO_4_, and ZnSO_4_ strongly inhibited enzyme activity. The HSDLb1 exhibited high specificity ketonization of the –O-H group attached to the C3 position of C-K, and converted C-K → 3-oxo-C-K. The proposed C-K pathway was suggested by TLC, HPLC and LC/MS data analysis as shown in Figure 5. Additionally, 3-oxo-C-K showed no cytotoxicity, strongly inhibits the MMP-1 activity and increased the production of procollagen type 1 than ginsenoside C-K as shown in Figure 6A–C.

In current society, young or fresh looking skin is wanted by many people all over the world, and therefore, many persons like to buy chemical-based ingredients for fresh skin looking. Premature skin aging is caused by ultraviolet radiation (UV), also referred to as photoaging, which is a chronic skin disorder and different from chronologically skin aging. Relevant research studies found that UV irradiation accelerates overexpression of ROS, which boosts the activation of MMPs that break down collagen by activating AP-1 [40,41,42]. But most of the chemical-based ingredients cause skin allergies and irritation [43,44]. To avoid those chemical-based ingredients (or cosmetics products) for skin, many research studies have been focused on the development of plant-based ingredients or their derived compounds, which are considered as none or less toxic and free of adverse effects. Therefore, in oriental herbal medicine, red ginseng is a very popular health-promoting food but contains more than 90% of major ginsenosides (Rb1, Rb2, rc, Rd, rg1 and Re) and approximately less than 0.02% minor ginsenosides including ginsenoside C-K on dry weight. Although both major and minor ginsenosides show anti-inflammatory, anti-diabetic, antiaging, antioxidant, and neuroprotective properties but the extent of pharmacological effects are significantly high in minor ginsenosides when compared with major ginsenosides [8,9,10,11,12,13,14]. The minor ginsenosides (which are derived from major ginsenosides via bioconversion) are smaller in molecular weights and easily cross the cell membranes, resulting in good absorption in the bloodstream [43]. Therefore, many studies have been summarized the efficacy and usefulness of minor ginsenosides. Therefore, the interest of researchers increases toward the conversion of major ginsenosides to minor ginsenosides due to their high pharmacological effects, and almost all the previously researcher published data about the bioconversion of major ginsenosides to minor ginsenosides [15,16,17,18,19,20] with their pharmacological effects. However, there is no single study published to prove the further conversion of minor ginsenosides to another pharmacological active metabolite using recombinant enzyme. So, our major focus was to identify a novel gene for the conversion of minor ginsenoside C-K to another high pharmacological active compound with its initial screening as antiaging activity. 

Thus, in this study we found novel gene [3-β-hydroxysteroid dehydrogenases (HSDLb1)] from a probiotic (*Lactobacillus brevis* DCY65-1) species which belong to SDR family. The gene HSDLb1 was cloned and expressed in *E*. *coli* BL21 (DE3) in a soluble form and was characterized. It had optimum activity at 30 °C and pH 6.0–8.0. The recombinant HSDLb1 could convert C-K to another active metabolite 3-oxo-C-K through selective hydrolysis of -O-H group attached to C3 position of C-K as shown in Figure 5. 

As, minor ginsenoside C-K is a precursor compound of 3-oxo-C-K [17] that can effectively decrease the UVB-induced side reaction [45], antioxidant ability [24], and resist free radicals that cause skin photoaging [24]. Therefore, the efficacy of 3-oxo-C-K was screened out for the ultraviolet irradiated human fibroblast cell. Interestingly, we found that in vitro analysis the metabolite of C-K (3-oxo-C-K) shows more suppressed MMP-1 production activity and accelerates secretion of procollagen type I in HDFs (Figure 6B,C). Our results indicate that 3-oxo-C-K is a potential agent that can be used to protect against UVB-induced skin photoaging. In addition, we aimed that 3-oxo-C-K should be studied further as a novel photoaging ingredient. 

## 3. Materials and Methods

### 3.1. Chemical Reagents and Ginsenoside Standards 

Ginsenosides C-K was purchased from the Ginseng Genetic Center (Yongin-si, Republic of Korea) and 3-oxo-C-K was obtained by our research group as described by Jin et al. [17]. Other chemical regents such as acetonitrile, and methanol were purchased from SK Chemicals (Ulsan, Republic of Korea).

#### 3.1.1. Strain Isolation and Ketonization of Ginsenoside C-K

Kimchi is one of the most famous fermented pickle (made from vegetable sources) in south Korea and used as side dish. It is one of the rich sources for the *Lactobacillus* species, and more than 600 (LAB) strains were isolated from Kimchi collected from numerous regions in Korea [40]. Therefore, during the screening of *Lactobacillus* species in Kimchi (Korean fermented pickle), the Kimchi samples were collected from a local market in Yongin city, Korea. The sample was carefully dissolved in 0.80% sterilized saline, serially diluted, spread on MRS agar medium (Difco, Franklin Lakes, NJ, USA), and incubated at 37 °C for seven days. After seven days of incubation, single colony was obtained by subculture on MRS agar medium. Strain DCY65-1 was sought out (using 16S rRNA sequence analysis) and regularly cultured on MRS (De Man, Rogosa and Sharpe) agar medium at 37 °C and preserved in 25% glycerol (*v*/*v*) stock vial at −80 °C [17].

The initial ketonization of C-K was carried out as described previously [17] with a slight modifications. Briefly, the strain DCY65-1 at its maximum growth phase was added with 600 μL of 1000 ppm C-K in a 1.5 mL tube and incubated at 37 °C for 24 h in shaking incubator. After 24 h, a 100 μL aliquot was take out and mixed with 100 μL of water-saturated n-butanol (upper layer), and centrifuged. After centrifugation, the upper layer was analyzed by thin layer chromatography (TLC) [16,17].

#### 3.1.2. Gene Cloning, Expression and Purification of Recombinant HSDLb1

As the strain *L. brevis* DCY65-1 was positive for the ketonization of minor ginsenosides F1 and C-K [28], therefore the genomic DNA of DCY65-1 was extracted for target gene identification and cloning. The extracted DNA was purified using a DNA isolation kit (GeneAll, Seoul, Republic of Korea) and the target gene (encoded 3-β-HSD) was amplified by PCR using the *Pfu* polymerase enzyme (GeneAll, Republic of Korea). After amplification, the PCR product was washed and introduced into the pMAL-c5X fusion vector (the ampicillin resistance gene *bla* in pMAL-c5X, *HSDLb1* gene was cloned into plasmid pMAL-c5X under control of promoter tac. The multiple cloning site (MCS) is positioned to allow translational fusion of the E. coli maltose binding protein (MBP) to the N-terminus of the cloned target protein. The affinity of MBP to maltose was used to purify the fusion protein) using an EzCloning Kit (Enzynomics Co., Ltd., Daejeon, Korea). *Nde*I and *Eco*RV were used as restriction sites. The resultant HSDLb1 gene was transformed into *E. coli* BL21 (DE3), and recombinant *E. coli* (comprising a plasmid containing the *HSDLb1* gene) was grown in LB-ampicillin (Luria-Bertani medium containing 100 mg/mL ampicillin) medium at 37 °C. When the culture attained an OD_600_ (optical density at a wavelength of 600 nm) value of 0.4–0.6, then the gene was expressed by adding 0.3 mM isopropyl-β-d-thiogalactopyranoside (IPTG). After adding IPTG, the culture was again incubated at 28 °C in shaking incubator for 9 h. Next day, the pellets were collected via centrifugation (at 5000× *g* for 20 min at 4 °C), and washed with 20 mM sodium phosphate buffer (pH 7.4). After washing, the cells were centrifuged, re-suspended in 20 mM phosphate buffer and the cell suspension was sonicated using a Branson Digital Sonifier [70% power, 400 W, Emerson Electric Co., Danbury, CT, USA]. After sonication, the cells debris was removed via centrifugation (at 16,000× *g* for 15 min) and the recombinant protein [MBP-tagged (maltose binding protein)] was purified through a small column [(containing amylose resin) New England BioLabs, Ipswich, UK]. Recombinant protein uniformity was measured by 12% SDS-PAGE (Sodium Dodecyl Sulphate-Polyacrylamide Gel Electrophoresis) analysis [7,16] using a staining solution (Coomassie Blue).

#### 3.1.3. Effect of Different pH, Temperature, and Metal Ions

Generally, enzymes are very sensitive to low or high pH values and the temperature of their surroundings [15,16,17,18]. Thus, very low or very high pH values effect in broad loss of enzymatic activity. Therefore, the effect of pH on the activity of HSDLb1 was evaluated using 5.0 mM C-K as a substrate in 20 mM of different buffers solutions [KCl-HCl and glycine-HCl for pH 2–3, sodium acetate and sodium phosphate (pH 4.0–7.5), Tris-HCl (pH 8.0, and 10.0), and Tris-NaOH (pH 12.0)] for 24 h. To examine the effect of temperature on HSDLb1 activity the samples were kept at the optimum pH value for 24 h in 100 mM potassium phosphate buffer containing 5.0 mM C-K at various temperature (20, 25, 30, 37, 40, 45, 50, 55, and 60 °C) for 24 h. After 24 h, the samples were collected and HSDLb1 enzymatic activity was identified. Additionally, the effects of metal ions on the activity of HSDLb1 were also identified. Briefly, 5.0 mM C-K as a substrate in 10 mM (final concentration) of CoCl_2_, MgCl_2_, FeCl_3_, NaCl, CuSO_4_, NH_4_Cl, KCl, CaCl_2_ or ZnSO_4_ was mixed with 100 µL recombinant HSDLb1 and the samples were incubated for 30 min at 30 °C. The results were compared with those of the control (HSDLb1 enzyme with 5.0 mM C-K but without metal ions). All these experiments were performed in triplicate and the results were determined by TLC. Image J software (1.53d, NIH, Bethesda, MA, USA) was used to calculate the strip conversion rate.

### 3.2. TLC, HPLC and LC/MS Analysis

The bioconversion of C-K to 3-oxo-C-K was identified using Silica Gel 60 (TLC) plates. The spots were developed by dipping the plates in CHCl_3_-CH_3_OH-H_2_O solution [65:35:10 (*v*/*v*/*v*)]. After dipping the plates, the plates were taken out and dried with an air dryer, and the spots were noticed by spraying 10% (*v*/*v*) H_2_SO_4_ dissolved in water and heating at 110 °C for 5–10 min [28]. Additionally, the sample (C-K and 3-oxo-C-K) peaks were identified by HPLC. C18 column (50 × 4.6 mm, 2.6 μm) with two solvents, DDW [deionized distilled water (solvent A)] and acetonitrile (solvent B) at A/B ratios of 81:19, 81:19, 71:29, 71:29, 60:40, 44:56, 30:70, 10:90, 10:90, 81:19, and 81:19; with run times of 0–7, 7–11, 11–14, 14–25, 25–28, 28–30, 30–31.5, 31.5–34, 34–34.5, and 34.5–40 min, respectively. The flow rates of the solvents were adjusted to 600 µL min^−1^ with a detection wavelength of 203 nm. Furthermore, the bioconversion of C-K to 3-oxo-C-K was confirmed by LC/MS (liquid chromatography–mass spectrometry) using an Agilent QQQ/MS (triple quadrupole mass spectrometer) instrument with positive polarity and an ion trap analyzer. 

### 3.3. In Vitro Cell Viability Analysis

Human dermal fibroblast (HDF) cells were obtained from local cell line bank of Korea [Korean Cell Line Bank (KCLB)]. The HDF cell were grown and maintained in Dulbecco’s modified Eagle’s medium (DMEM) supplemented with 10% heat inactivated fetal bovine serum (FBS) and penicillin/streptomycin (100 U/mL) at 37 °C in 5% CO_2_ [25].

#### 3.3.1. Ultraviolet Irradiation

To determine the effect of ultraviolet irradiation on the HDFs, the HDFs (fibroblast cells) were seeded at a density of 4 × 10^4^ cells/mL and allowed to attach overnight. To emit ultraviolet irradiation, a halide lamp (UVASUN 3000) was used as a source of Ultraviolet irradiation. The emitted radiation with a wavelength of 340–450 nm. The next morning, the cells were removed and washed twice with a buffer solution (PBS), and the medium was replaced with 1 mL of fresh PBS. The incident dose was 66 mW/s at the surface of the HDFs. Beckman spectrophotometer (Beckman UV 5270, Beckman, Munich, Germany) was used for spectral distribution of UV lamp [halide lamp (UVASUN 3000, Mutzhas, Munich, Germany)] [41,42,46].

#### 3.3.2. Assay for Inhibition of Matrix Metalloproteinase-1 (MMP-1) Expression and Production of Procollagen Type I

After sample treatment, 1 mL of supernatant was collected from each well, and the secretion of MMP-1 and procollagen type I in the medium were analyzed with commercially available ELISA kits following the manufacturer’s instructions [41,42,46].

#### 3.3.3. Cytotoxicity of Ginsenoside C-K and 3-oxo-C-K

To determine the cytotoxicity of ginsenoside C-K and 3-oxo-C-K on the human dermal fibroblasts cells, the cells were cultured in 96-well plates at a density of 1 × 10^4^ cells/well. The plates were incubated at 37 °C in 5% CO_2_ for 24 h. After 24 h, the medium was replaced with fresh medium contained different concentrations of C-K and 3-oxo-C-K, and the plates were further incubated for 24 h. The cytotoxicity or cell viability of C-K and 3-oxo-C-K was measured by the MTT [3-(4,5-dimethylthiazol-2-yl)-2,5-diphenyltetrazolium bromide] assay with small changes [25]. Briefly, 10 µL of solution [MTT (5 mg/mL)] was added to each well, and the plates were re-incubated for 4 h. After 4 h, the solution was removed and cells were mixed or lysed with 100 µL DMSO, and the absorbance was measured at 570 nm [41,42,46].

## 4. Conclusions

To the best of our knowledge, this is the first report on the use of recombinant enzyme (HSDLb1) treatment for conversion of C-K to 3-oxo-C-K. This study also demonstrates the pharmacological effect of 3-oxo-C-K in laboratory for MMP-1 and procollagen type 1 activities analysis. Thus, in vitro study the 3-oxo-C-K show no cytotoxicity and strongly inhibit the collagenase (MMP-1) activity and boost the production of procollagen type 1 compared to C-K. Thus, we suggest that, 3-oxo-C-K may be a potential pharmacologically active compound than C-K and can be used in various cosmetic products for the inhibition of UV radiation and photoaging agent, for which further study will be needed.

## Figures and Tables

**Table 1:** Effect of 10 mM metal ions on the recombinant HSDLb1 activity.

Metal Ions or Reagents	Relative Activity ± SD (%) 10 mM
Control	100.0 ± 5.4
CoCl_2_	19.8 ± 1.0
MgCl_2_	138.1 ± 8.5
FeCl_3_	15.6 ± 1.3
NaCl	85.6 ± 6.2
CuSO_4_	10.6 ± 0.5
NH_4_Cl	89.4 ± 5.1
KCl	76.7 ± 6.0
CaCl_2_	63.4 ± 3.4
ZnSO_4_	6.5 ± 0.9

## Data Availability

Not applicable.

## Supplementary Materials

The following supporting information can be downloaded at: https://www.mdpi.com/article/10.3390/molecules28093792/s1, Figure S1: Phylogenetic tree showing the evolutionary status of *L. brevis* 3-β-HSD. The tree was constructed by using the maximum likelihood method based on amino acid sequence analysis (amino acid sequences including accession numbers were obtained from the NCBI and UniProt database). Bootstrap values (expressed as percentages of 1000 replications) greater than 40% are shown at branch points. Bar 0.5 substitutions per nucleotide position.
