# Supplementary material for: Ketonization of Ginsenoside C-K by Novel Recombinant 3-β-Hydroxysteroid Dehydrogenases and Effect on Human Fibroblast Cells
Source: Molecules. 2023 Apr 28;28(9):3792. doi: 10.3390/molecules28093792 (PMC10180105; doi:10.3390/molecules28093792)
Supplement: Supplementary file 1 [file molecules-28-03792-s001.zip › molecules-2224332-supplementary.pdf]

Supplementary data file

Ketonization of ginsenoside C-K by novel recombinant 3-β-hydroxysteroid dehydrogenases and effect on human fibroblast cells

Yan Jin[1], Dandan Wang[2], Wan Taek Im[3,4], Muhammad Zubair Siddiqi[3*] and Deok-Chun Yang[4*]

[1] School of Life Science, Nantong University, Nantong 226019, China
[2] College of Life Sciences, Yantai University, Yantai 264005, China
[3] Department of Biotechnology, Hankyong National University, 327 Jungang-ro Anseong-si, Gyeonggi-do 17579, Republic of Korea
[4] Department of Oriental Medicinal Material & Processing, College of Life Science, Kyung Hee University, Seocheon-dong, Giheung-gu, Yongin-si, Gyeonggi-do, Republic of Korea
* Correspondence: mzsiddiqi1988@gmail.com (M.Z.S.); dcyang@khu.ac.kr (D.-C.Y.)

Figure S1

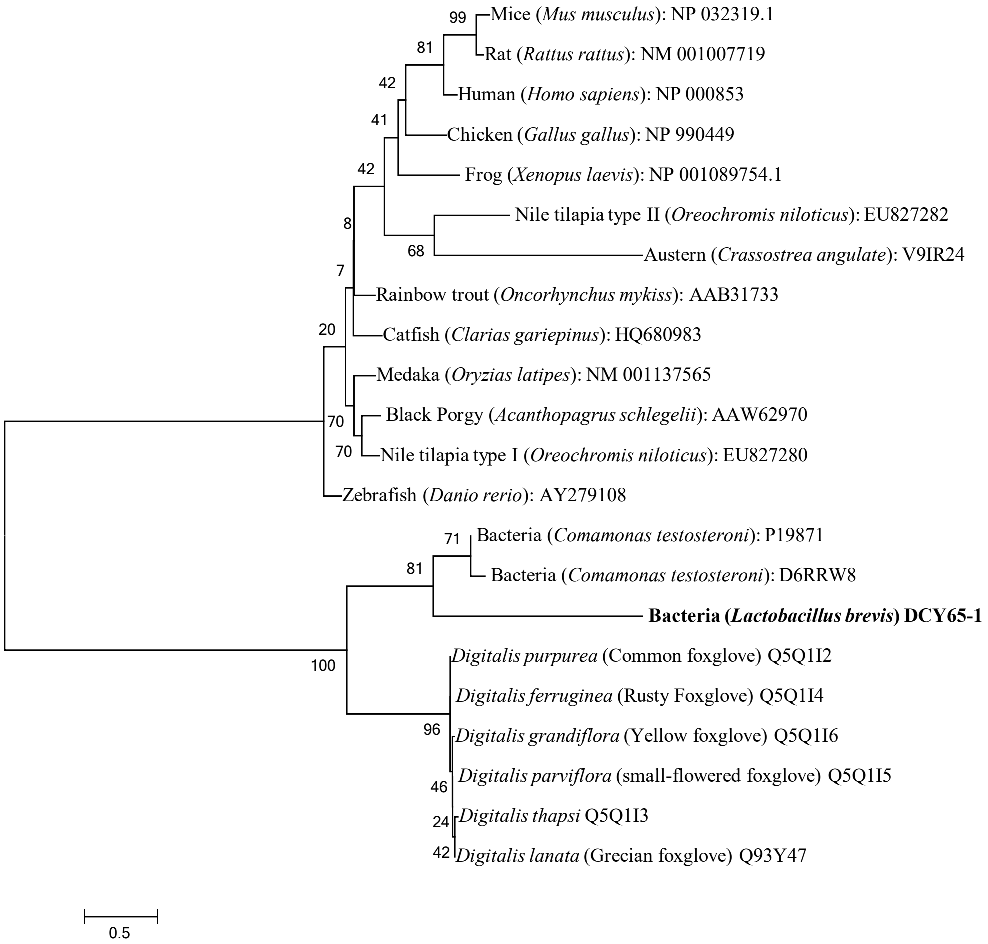

Figure S1. Phylogenetic tree showing the evolutionary status of *L. brevis* 3-β-HSD. The tree was constructed by using the maximum likelihood method based on amino acid sequence analysis (amino acid sequences including accession numbers were obtained from the NCBI and UniProt database). Bootstrap values (expressed as percentages of 1000 replications) greater than 40 % are shown at branch points. Bar 0.5 substitutions per nucleotide position.
